# Artificial intelligence assisted genomic profiling of ovarian cancer liver metastases

**DOI:** 10.3389/fgene.2026.2004540

**Published:** 2026-10-20

**Authors:** Louxun Sun, Zhongshan Li, Shiming Chen

**Affiliations:** Department of Hepatobiliary Pancreatic Surgery, Shaoxing Second Hospital, Shaoxing, P. R. China

**Keywords:** artificial intelligence, genomic medicine, germline susceptibility, homologous recombination deficiency, liver metastasis, ovarian cancer, pharmacogenomics, spatial multi-omics

## Abstract

Liver involvement in ovarian cancer is often treated as a single clinical entity, although liver-surface implants and parenchymal deposits occupy different anatomical settings and may not share the same molecular history. Primary-tumor profiling is informative, but can miss lesion-specific evolution, treatment-associated cell states, and microenvironmental structure. This Mini Review examines how germline susceptibility, somatic change, spatial biology, and artificial intelligence might be combined in high-grade serous ovarian cancer. The central task is not simply outcome prediction. It is to distinguish constitutional from tumor-acquired variation, retain lesion identity and treatment timing, and relate molecular programs to defined spatial compartments. Current data support marked clonal and microenvironmental heterogeneity in ovarian cancer, but direct molecular evidence from hepatic lesions remains sparse. AI-based integration is therefore best viewed as a way to formulate and test reproducible hypotheses, rather than as a validated route to select liver-directed therapy. Paired sampling, orthogonal assays, patient-level validation, and decision-specific clinical endpoints will be needed before these models can influence management.

## 1 Introduction

Liver involvement in ovarian cancer is not fully described by the single label ‘liver metastasis.’ FIGO staging distinguishes implants on the liver surface from parenchymal metastases (Renz et al., 2025). The distinction matters clinically, but it does not tell us whether the lesions share clonal ancestry, DNA-repair status, or treatment-associated states (Renz et al., 2025). Germline susceptibility provides constitutional context. Somatic mutations, copy-number change, methylation, and transcriptional programs describe the tumor as it changes over time. These layers can intersect, but they answer different questions. A primary-tumor sample is therefore a useful starting point, not necessarily a complete representation of liver disease.

Matched, multiregion studies have made this limitation more tangible. Sequencing of primary high-grade serous ovarian cancer (HGSOC) and distant lesions has shown branching evolution and incomplete sharing of alterations between sites (Masoodi et al., 2020). In treatment-naive HGSOC, mutational processes and anatomic location have also been associated with different immune states (Vázquez-García et al., 2022). Spatial transcriptomic work linked ovarian cancer subclones to copy-number patterns and local stromal or immune neighborhoods (Denisenko et al., 2024). A recent atlas further associated tumor-cell MHC class II expression with spatial immune organization and outcome (Perez-Villatoro et al., 2026). None of these studies, however, establishes a recurrent molecular program specific to ovarian cancer liver lesions.

Artificial intelligence becomes relevant when data from these different levels must be interpreted together. Multimodal machine-learning models have improved prognostic stratification in HGSOC cohorts (Boehm et al., 2022), yet a prognostic score is not automatically a model of hepatic dissemination or treatment benefit. A clinically useful system would need to retain the origin of the genomic signal, lesion identity, treatment exposure, and sampling time. Losing those labels can produce a technically impressive model with little biological meaning.

This review considers ovarian cancer as a reproductive-system malignancy in which inherited susceptibility and tumor evolution can be studied together. Its focus is deliberately narrow: what genomic evidence, validation, and endpoint definition would be needed before AI-assisted molecular information could be used alongside anatomy and treatment history in liver-directed management?

This narrative Mini Review prioritizes primary HGSOC studies of genomic evolution, treatment-associated states, and spatial organization, together with relevant computational methods and clinical trials. Evidence from ovarian cancer hepatic lesions is distinguished from broader ovarian cancer observations and cross-cancer mechanisms; the latter inform hypothesis development. Representative studies are selected for their relevance to these questions.

## 2 Germline, somatic, and lesion-specific genomic heterogeneity

Primary and metastatic tumors may share a clonal origin while diverging in ways that matter for interpretation. The first step is to separate inherited variants from alterations detected only in tumor tissue. A multiregion whole-exome study examined 33 tumor regions from six patients with HGSOC, including two patients with liver metastases (Masoodi et al., 2020). Most distant metastases were sampled after chemotherapy, so anatomical and temporal differences need to be considered together. Some lesions retained much of the primary tumor’s mutational history, whereas others appeared to disseminate earlier and evolve in parallel. The hepatic observations provide direct, small-cohort evidence of metastatic evolution, without defining a recurrent liver-metastasis program. A variant found in one metastatic lesion remains a candidate site-associated change until its distribution and function have been tested.

Copy-number architecture adds a further source of diversity. A pan-cancer analysis identified chromosomal-instability signatures that help organize otherwise complex structural changes (Drews et al., 2022). Whole-genome sequencing of 640 ovarian tumors from 243 patients subsequently identified five HGSOC subtypes, including homologous-recombination-deficient and proficient groups with different transcriptional programs and treatment responses (Micoli et al., 2025). Germline findings concern inherited risk; tumor homologous recombination deficiency (HRD) and treatment-response features concern the biology of an established cancer. They should be interpreted in their own clinical context (Renz et al., 2025; Micoli et al., 2025; Swisher et al., 2021).

Treatment can blur comparisons across sites. In ARIEL2, loss of *BRCA1* promoter methylation was associated with resistance, whereas genomic scars related to HRD could remain detectable after platinum resistance had emerged (Swisher et al., 2021). At the level of DNA sequence and copy number, BriTROC-1 found broad concordance in mutations and copy-number profiles between diagnosis and relapse, without a common acquired copy-number explanation for chemotherapy resistance (Smith et al., 2023). Longitudinal single-cell RNA sequencing still identified enrichment of a stress-associated state during treatment, with accompanying stromal changes (Zhang et al., 2022). These findings address complementary biological levels: constitutional variants describe inherited background, tumor-acquired alterations describe clonal architecture, genomic scars record accumulated damage, and transcriptional profiles capture the sampled cellular state. An HRD-associated scar can persist despite restoration of repair-related function, as illustrated by *BRCA1* demethylation in ARIEL2 (Swisher et al., 2021). A lesion may therefore remain genetically similar yet behave differently under therapy.

For liver-focused studies, matched non-tumor DNA, primary-tumor, hepatic, and nonhepatic samples would allow inherited, shared, and acquired events to be evaluated separately. Lesion type and treatment timing should be recorded at collection. Surface implants and parenchymal deposits are unlikely to be interchangeable analytical units. With this design, AI can be used to test a defined hypothesis about site-associated biology rather than search indiscriminately for a liver-metastasis signature.

## 3 Spatial context and lesion identity in hepatic involvement

Anatomy is a sensible starting point for molecular interpretation, but it is not enough on its own. FIGO distinguishes liver-surface implants from parenchymal metastases (Renz et al., 2025). In genomic studies, the specimen annotation should go further: whether the tissue is a surface implant, direct extension, or a separate parenchymal deposit; its tumor purity; and the amount of adjacent hepatic tissue all affect interpretation. Location alone does not establish the route of dissemination or prove a shared molecular origin.

Spatial profiling adds information that bulk tumor genotyping cannot provide. Ovarian cancer subclones with different copy-number patterns have been linked to distinct local environments (Denisenko et al., 2024). Single-cell work has also described immune-infiltrated, immune-excluded, and immune-desert phenotypes, with differences in macrophage and fibroblast states and candidate chemokine axes (Hornburg et al., 2021). The MHC class II spatial atlas adds another level of association between tumor-cell state, immune organization, and outcome (Perez-Villatoro et al., 2026). These observations are important, although their hepatic relevance remains to be demonstrated directly.

The omentum illustrates why metastatic sites cannot simply be substituted for one another. Single-cell analysis of benign and metastatic human omentum described marked remodeling of mesothelial, progenitor, and immune populations (Lischetti et al., 2026). The adipose-rich omentum is not hepatic parenchyma. Similarly, liver-metastasis studies in other cancers identify mechanisms worth examining in ovarian cancer, rather than mechanisms to transfer uncritically. Monocyte-derived macrophages contributed to CD8-positive T-cell elimination through Fas-FasL signaling in one preclinical model (Yu et al., 2021). In colorectal cancer and melanoma models, depletion of tumor-reactive hepatic stellate cells reduced metastatic burden and stromal remodeling (Benedicto et al., 2025).

A useful hepatic specimen therefore preserves the tumor-liver interface and distinguishes malignant cells from neighboring hepatic and stromal compartments. Matched germline DNA and tumor DNA/RNA make it less likely that a lesion-specific somatic pattern is mistaken for a constitutional signal. Platinum exposure, PARP inhibitor use, and the interval between therapy and sampling are also part of the biological record.

The next question is whether particular copy-number or transcriptional programs recur in defined cellular neighborhoods. A ligand-receptor prediction or spatial adjacency provides a hypothesis, not proof of signaling. Reproducibility across patients and lesion classes is a more useful test than a single high-scoring pattern. Figure 1 separates mechanisms reported in other cancer models (Yu et al., 2021; Benedicto et al., 2025) from ovarian cancer spatial evidence (Denisenko et al., 2024; Perez-Villatoro et al., 2026) and from hypotheses that still need hepatic validation.

**FIGURE 1:**
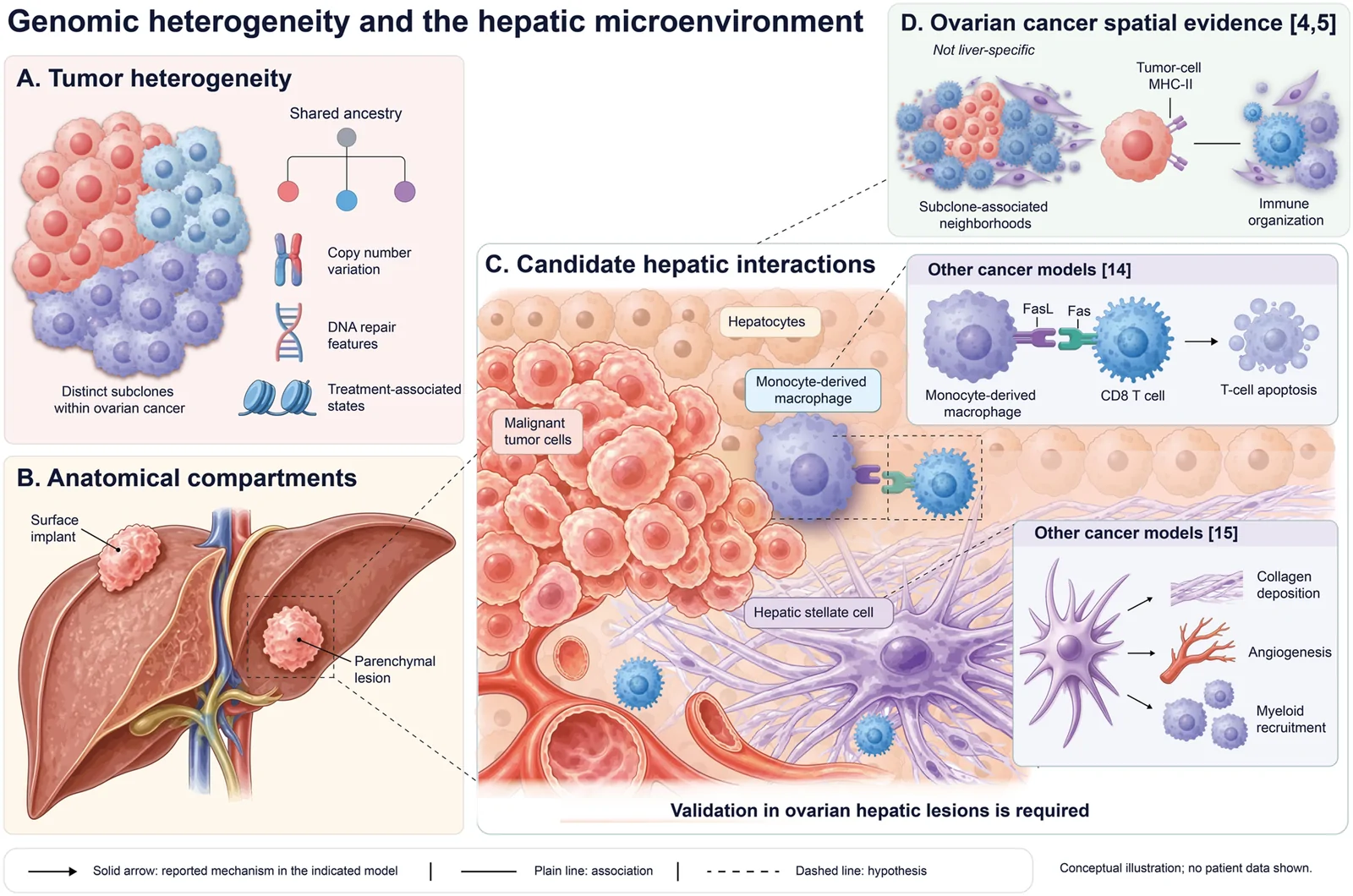
Genomic heterogeneity and the hepatic microenvironment in ovarian cancer. **(A)** Shared clonal ancestry, copy-number variation, DNA-repair features, and treatment-associated cell states in ovarian cancer. **(B)** Liver-surface implants and parenchymal lesions are shown as distinct anatomic compartments; location alone does not define the route of dissemination. **(C)** Candidate interactions at the tumor-liver interface. Macrophage-mediated Fas-FasL-dependent CD8-positive T-cell apoptosis and hepatic stellate cell-associated collagen deposition, angiogenesis, and myeloid recruitment are supported by studies in other cancer models and require direct validation in ovarian cancer hepatic lesions. **(D)** Ovarian cancer spatial studies link tumor subclones and tumor-cell MHC class II expression to local cellular organization, without establishing liver-specific relationships. Solid arrows indicate reported mechanisms in the specified models, plain lines indicate associations, and dashed connections indicate hypotheses. All tissue and clonal patterns are schematic. MHC-II, major histocompatibility complex class II.

## 4 AI-assisted integration of genomic and spatial evidence

AI need not be framed as a generic therapeutic assistant. Its practical role in this setting is to connect molecular measurements to a clearly defined biological question while retaining patient identity, lesion class, and sampling time. Variation between samples can arise from clonal ancestry, germline background, treatment exposure, tissue composition, or technical processing. These sources are not interchangeable. A model that loses them may learn a batch effect or a patient-specific pattern rather than the biology of a hepatic lesion.

One task is to ask whether a transcriptional program tracks a structural alteration or reflects a reversible state within a related clone. MOFA + offers a factor-based approach to variation across molecular modalities (Argelaguet et al., 2020). GLUE relates chromatin accessibility and gene expression through a guidance graph (Cao and Gao, 2022), and MultiVI can model paired and unpaired multimodal data when one assay is missing in some cells (Ashuach et al., 2023). These methods can organize evidence, but alignment, imputation, and proximity in latent space remain computational observations. RNA-inferred copy-number change should be separated from DNA-confirmed alteration, especially where claims concern inherited predisposition or treatment selection.

Spatial methods shift attention from which states are present to where they occur. SpatialGlue integrates molecular and spatial relationships with a graph neural network (Long et al., 2024); SMART extends graph-based integration across spatial multi-omic sections (Du et al., 2026). In hepatic lesions, such approaches could identify domains at the tumor-liver interface and compare them with morphology. Yet a graph edge is not biological communication, and smoothing can obscure small invasive populations or blur the tumor-liver boundary. Domains defined computationally should be checked against pathology and, where feasible, independent measurements.

Foundation models may help when disease-specific cohorts are small. scGPT was developed to learn transferable single-cell representations (Cui et al., 2024), and Nicheformer combines dissociated and spatial transcriptomic data to model cellular context (Tejada-Lapuerta et al., 2025). Transfer to treated ovarian cancer liver metastases remains an empirical question. A carefully annotated, modest-sized cohort may be more informative than a larger dataset with uncertain lesion identity.

For example, a study could ask whether a treatment-associated tumor-cell program is enriched at the tumor-liver interface. Pathology would define the interface and tumor compartments, while treatment records would establish sampling context. Matched tumor and non-tumor DNA would distinguish somatic from constitutional variation. Depending on available assays, MultiVI could integrate paired or unpaired RNA and chromatin-accessibility measurements (Ashuach et al., 2023), while a spatial method could delineate domains from spatially resolved modalities (Long et al., 2024; Du et al., 2026). These are task-dependent choices, not obligatory sequential steps. The output would be a candidate program whose localization should be checked by RNA or protein assays and whose enrichment should recur across independent patients after considering cellular composition and treatment exposure. The added value of integration should also be assessed against a single-modality analysis or a prespecified expression score.

Existing ovarian cancer studies point to a restrained path forward. A spatial atlas used random forests to identify tumor-cell MHC class II expression, followed by complementary molecular and functional analyses (Perez-Villatoro et al., 2026). Another study combined clinicogenomic, pathological, and radiological data for prognostic stratification (Boehm et al., 2022). Both support the value of integration; neither is a validated model for choosing liver-directed treatment. Clinicogenomic input also cannot be assumed to provide germline variant interpretation.

Validation must match the intended claim. Discovery work needs replication of a clone-niche relationship across patients while accounting for treatment exposure and specimen composition. For prediction in new patients, all lesions, sections, cells, and time points from each patient should remain in one data partition; cellular observations do not substitute for independent patients. Data-dependent preprocessing, feature selection, and tuning should be restricted to development data or training folds, with evaluation data kept separate. Performance should be compared with clinical and pathological baselines, reporting calibration, uncertainty, and external discrimination (Collins et al., 2024; Moons et al., 2025). TRIPOD + AI addresses reporting, whereas PROBAST + AI supports quality and risk-of-bias appraisal; neither replaces independent evaluation. Biological replication, predictive performance, and comparative evidence of treatment benefit answer different questions. Figure 2 summarizes this pathway from sampling to endpoint-specific assessment.

**FIGURE 2:**
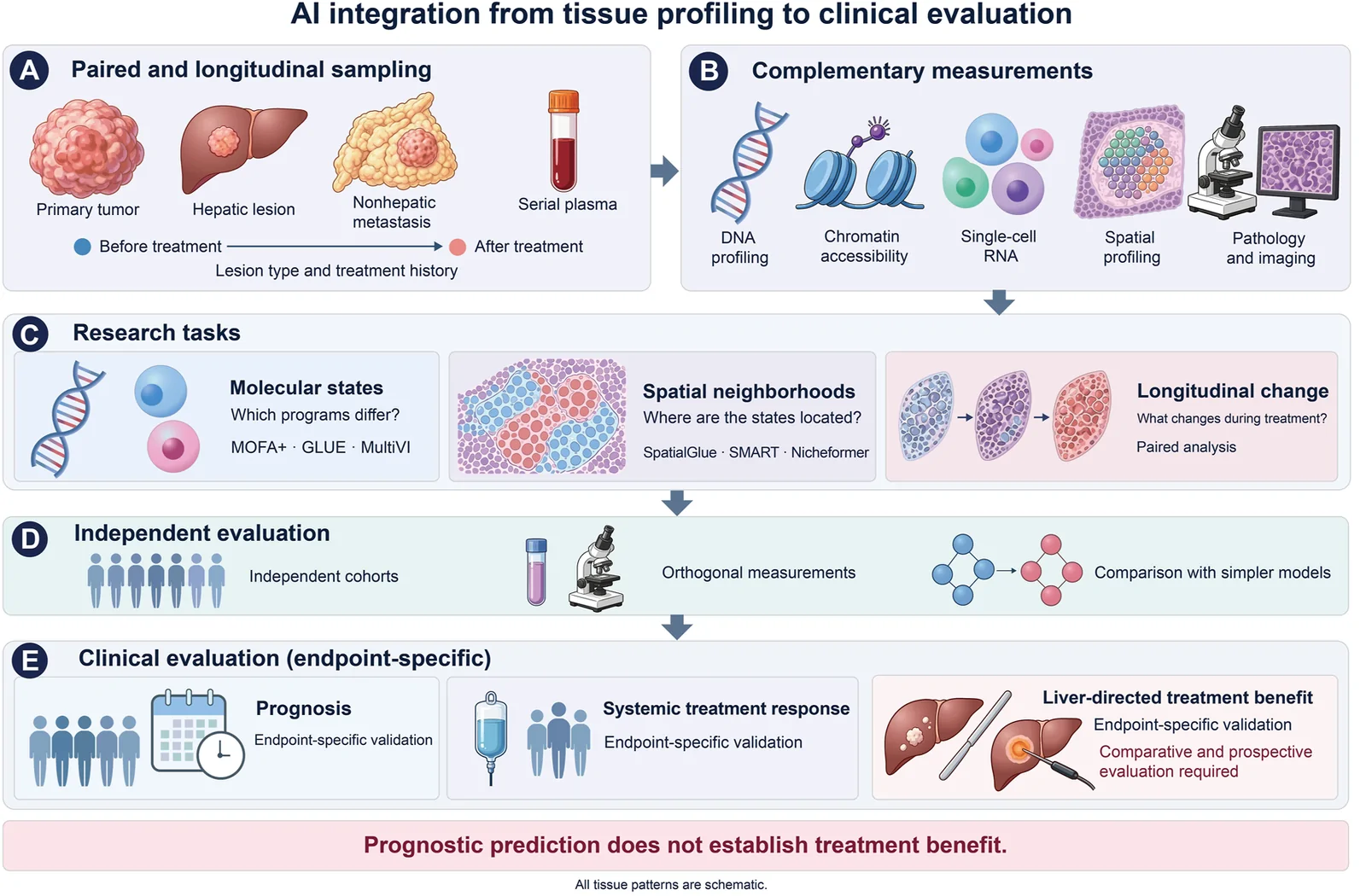
AI integration from tissue profiling to clinical evaluation in ovarian cancer liver metastases. **(A)** Paired sampling of primary tumors, hepatic lesions, and nonhepatic metastases, supplemented by serial plasma collection and documentation of treatment timing. **(B)** Complementary measurements include DNA profiling, chromatin accessibility, single-cell RNA, spatial profiling, pathology, and imaging; not every modality is required or available for each specimen. **(C)** Analytical tasks include molecular-state comparison, spatial-neighborhood analysis, and longitudinal change. The listed methods are illustrative and are not validated components of a single liver-metastasis model. **(D)** Independent cohorts, orthogonal measurements, and comparison with simpler models are needed before clinical interpretation. **(E)** Prognosis, systemic-treatment response, and benefit from liver-directed therapy require endpoint-specific validation. Treatment benefit additionally requires comparative and prospective evaluation; prognostic accuracy alone is insufficient. All tissue patterns are schematic. AI, artificial intelligence.

## 5 Clinical interpretation in liver-directed management

Molecular information becomes clinically useful only when it addresses a defined decision. Resectability, extrahepatic disease, the prospect of complete cytoreduction, and prior systemic treatment remain the immediate considerations in ovarian cancer with liver involvement (Renz et al., 2025). A nationwide German analysis documented hepatic resection in 1,273 patients between 2020 and 2024 (Acs et al., 2025). It shows that liver surgery is part of contemporary practice, but administrative data cannot establish a survival benefit or identify a molecular subgroup that benefits more from resection.

Trials of secondary cytoreduction provide related, but not liver-specific, evidence. DESKTOP III reported longer overall survival with surgery followed by chemotherapy in selected patients with platinum-sensitive first relapse and a positive AGO score (Harter et al., 2021). In SOC-1, the intention-to-treat analysis did not cross its prespecified overall-survival threshold, whereas a crossover-adjusted analysis favored surgery (Jiang et al., 2024). GOG-0213 did not demonstrate a significant overall-survival advantage for secondary cytoreduction (Coleman et al., 2019). Selection differed: DESKTOP III used the AGO score, SOC-1 used iMODEL with imaging assessment, and GOG-0213 relied on investigator-assessed resectability. Complete resection, systemic-treatment exposure, and crossover also differed across trials (Harter et al., 2021; Jiang et al., 2024; Coleman et al., 2019); these comparisons cannot identify a single explanation for the divergent outcomes. None isolated hepatic resection or evaluated genomic criteria for selection. Their findings support context-specific assessment rather than generalization to all patients with resectable liver disease.

*BRCA1/2* status and HRD make the distinction between prognosis, systemic therapy response, and local-treatment benefit especially clear. SOLO1 reported durable benefit from maintenance olaparib in patients with BRCA-mutated advanced ovarian cancer, although the 7-year overall-survival analysis did not meet the prespecified significance threshold (DiSilvestro et al., 2023). PAOLA-1 did not show a significant overall-survival advantage in the intention-to-treat population, while prespecified exploratory analyses supported benefit in the HRD-positive subgroup (Ray-Coquard et al., 2023). These results inform systemic therapy. They do not establish a genomic rule for hepatectomy.

The same restraint applies to other liver-directed interventions. A 2026 case report described prolonged hepatic control after sequential yttrium-90 radioembolization alongside systemic therapy and other treatments (Wahl et al., 2026). The contribution of radioembolization cannot be separated in a single case. Likewise, a SEER-based machine-learning study modeled liver metastasis at diagnosis and overall survival from clinical and demographic variables, without genomic input (Li et al., 2026). Such models may be informative for prognosis, but they do not estimate the benefit of a specific local procedure.

For clinical translation, AI should report separate outputs for disease distribution, prognosis, systemic-treatment response, and estimated benefit from local treatment. The last of these requires a comparative framework. At present, AI-integrated genomic profiling is better placed to refine research stratification and multidisciplinary discussion than to make an independent decision about liver-directed therapy.

## 6 Discussion and future directions

The evidence supports a lesion-aware approach to ovarian cancer involving the liver. Genomic heterogeneity is well documented, and the hepatic microenvironment is a plausible additional source of variation. The unresolved problem is attribution. Many candidate mechanisms come from other tumor types or from ovarian cancer samples collected outside the liver. They are useful for designing studies, but they are not yet a hepatic genomic classifier.

Better sampling may prove as important as more elaborate computation. A recent ovarian cancer resource combined tumor tissue, ascites, and blood and illustrated meaningful differences among specimen types (Jacob et al., 2026). For hepatic disease, the most informative design would pair the primary tumor with a hepatic lesion and, where possible, a nonhepatic metastasis. Blood-derived DNA supplies constitutional context; serial plasma can contribute information about temporal change (Pascual et al., 2022). Surface implants and parenchymal deposits should remain distinct analytic groups.

Analytical sophistication must be matched by testing. Atlas-level benchmarking demonstrates the trade-off between removing batch effects and preserving biological variation (Luecken et al., 2022). In hepatic specimens, aggressive correction could erase a true site-associated state, whereas insufficient correction can manufacture one. A zero-shot evaluation found that simpler methods outperformed some single-cell foundation models for particular tasks (Kedzierska et al., 2025). Cell-cell communication inferences, including those generated with LIANA+, also need orthogonal support (Dimitrov et al., 2024). Spatial colocalization, protein-level assays, and perturbation experiments provide progressively stronger evidence.

The standard rises further when a model is proposed for treatment selection. Observational studies may emulate a target trial when randomization is not initially feasible, provided eligibility, treatment strategies, time zero, and causal estimands are specified explicitly. The TARGET statement offers reporting guidance for this work (Cashin et al., 2025). Early clinical evaluation should ask whether AI-supported recommendations change multidisciplinary decisions, introduce errors, or delay treatment; DECIDE-AI is useful in that setting (Vasey et al., 2022). Later trials need transparent protocols and reporting for both the AI intervention and clinician interaction, as set out in SPIRIT-AI and CONSORT-AI (Cruz Rivera et al., 2020; Liu et al., 2020).

In ovarian cancer models, circSERPINE2 was reported to promote platinum resistance through the miR-513a-5p/miR-526b-3p/SEMA7A axis, with accompanying changes in IL-6 signaling and macrophage polarization (Niu et al., 2026). A recent hepatocellular carcinoma study identified circRPPH1-mediated repression of *PPARα* through miR-7845-5p/SOX4 (Ru et al., 2026). These findings provide specific candidates for circRNA–miRNA–mRNA network analysis, although their relevance to ovarian cancer liver metastases has not been established.

AI methods could help prioritize additional interactions for investigation. HCLAMCMI, for example, predicts circRNA–miRNA interactions using hypergraph neural networks and contrastive learning (Chen et al., 2025). Combining such predictions with mRNA profiles could guide comparisons of ovarian and hepatocellular carcinoma networks to examine shared and disease-specific regulatory modules. The biological differences between the two cancers would need to be considered explicitly. Candidate relationships should then be tested in samples from ovarian cancer hepatic lesions through localization studies, binding assays, and perturbation-rescue experiments. Prediction scores alone would not establish a causal mechanism or treatment relevance.

A feasible first study would combine consistent lesion classification and treatment annotation with pathology and molecular assays chosen for a defined question. Additional spatial profiling can be introduced where tissue availability and assay quality permit. Candidate relationships should progress to independent patient-level replication and orthogonal testing before decision-focused clinical evaluation. The near-term value of AI is disciplined integration: a reproducible molecular state within a defined hepatic cellular neighborhood would already be meaningful. Whether that information improves a clinical decision and patient outcomes requires a subsequent comparative evaluation.
